# Long-Term Prediction Model for Hepatocellular Carcinoma in Patients with Chronic Hepatitis B Receiving Antiviral Therapy: Based on Data from Korean Patients

**DOI:** 10.3390/jcm11226613

**Published:** 2022-11-08

**Authors:** Ji Hun Lee, Seung Kak Shin, Seong Hee Kang, Tae Hyung Kim, Hyung Joon Yim, Sun Young Yim, Young-Sun Lee, Young Kul Jung, Ji Hoon Kim, Yeon Seok Seo, Jong Eun Yeon, Oh Sang Kwon, Soon Ho Um, Kwan Soo Byun

**Affiliations:** 1 Department of Medicine, Korea University College of Medicine, Seoul 02841, Korea; 2 Department of Internal Medicine, Gachon University Gil Medical Center, Gachon University College of Medicine, Incheon 21565, Korea; 3 Department of Internal Medicine, Inje University College of Medicine, Seoul 01757, Korea; 4 Department of Internal Medicine, Yonsei University Wonju College of Medicine, Wonju 26426, Korea; 5 Department of Internal Medicine, Korea University College of Medicine, Seoul 02841, Korea

**Keywords:** antiviral agent, hepatitis B virus, hepatocellular carcinoma, liver cirrhosis, risk factors

## Abstract

Predicting the development of hepatocellular carcinoma (HCC) is a key clinical issue in patients with chronic hepatitis B (CHB). The aim of this study was to develop a precise and simple HCC risk score for up to 10 years. A total of 1895 CHB patients treated with entecavir or tenofovir disoproxil fumarate were retrospectively recruited and randomized into derivation (*n* = 1239) and validation cohorts (*n* = 656). Variables proven to be independent risk factors for HCC in the derivation cohort were used to develop the prediction model. The ACCESS-HCC model included five variables (age, cirrhosis, consumption of ethanol, liver stiffness, and serum alanine aminotransferase). Areas under curves were 0.798, 0.762, and 0.883 for HCC risk at 3, 5, and 10 years, respectively, which were higher than those of other prediction models. The scores were categorized according to significantly different HCC incidences: 0–4, low; 5–8, intermediate; and 9–14, high-risk. The annual incidence rates were 0.5%, 3.2%, and 11.3%, respectively. The performance of this model was validated in an independent cohort. The ACCESS-HCC model shows improved long-term prediction and provides three distinct risk categories for HCC in CHB patients receiving antiviral therapy. Further research is needed for external validation using larger cohorts.

## 1. Introduction

Hepatocellular carcinoma (HCC) is one of the leading causes of death in patients with chronic viral hepatitis and the third most frequent cause of cancer-related deaths globally [1]. In patients with chronic hepatitis B (CHB), the risk of HCC increases by 5–100-fold that of those who do not have CHB [2]. Previous studies have demonstrated that antiviral treatment with nucleos(t)ide analogues, such as entecavir (ETV) or tenofovir disoproxil fumarate (TDF), can reduce the risk of HCC development, cirrhotic events, and mortality. ETV and TDF have been reported to be associated with 60–63% and 73–77% HCC risk reduction, respectively [3,4,5,6].

Before ETV and TDF were developed as antiviral agents, viral factors, such as hepatitis B virus (HBV) levels, were important predictive factors for the risk of developing HCC [7]. However, after the worldwide widespread of antiviral agents, the risk of HCC due to HBV has substantially decreased, resulting in other factors becoming more important [8]. The factors include lifestyle, status of underlying chronic liver disease, and baseline laboratory data. So far, most of these factors have been used for the short to intermediate (3 to 5 years) prediction of HCC. However, it is important to predict the long-term risk of HCC at the baseline follow-up before re-assessment during treatment.

Prediction of HCC development is difficult since it can occur even if the patient receives effective antiviral treatment, although the risk decreases with therapy [9,10,11,12]. The existing models that predict the risk of HCC in CHB patients, such as aMAP (age–male–ALBI (albumin-bilirubin score)–platelets score) [13], CAGE-B (cirrhosis and age score) [14], SAGE-B (stiffness and age score) [14], CAMD (cirrhosis, age, male sex, and diabetes mellitus model) [15], CAMPAS (cirrhosis on ultrasonography, age, male gender, platelet count, albumin, and liver stiffness model) [16], CU-HCC (Chinese University model) [17], GAG-HCC (guide with age, gender, HBV DNA, core promoter mutations and cirrhosis score) [18], PAGE-B (patients’ age, gender, and platelets score) [19], REACH-B (risk estimation for hepatocellular carcinoma in chronic hepatitis B score) [20], and REAL-B (real-world effectiveness from the Asia Pacific rim, liver consortium for HBV score) [21], provide information about the various important risk factors of HCC in CHB patients. However, CU-HCC, GAG-HCC, and REACH-B are currently less useful tools because these models were developed before the wide use of antiviral agents, such as ETV and TDF. In addition, the SAGE-B model requires additional evaluation at year 5 for the prediction of long-term HCC risk. Recently, it has been known that liver stiffness provides objective information regarding the status of liver fibrosis or liver cirrhosis, which are closely associated with the development of HCC [22,23]. However, only a few models included liver stiffness as a predictive factor in CHB patients receiving antiviral therapy.

The primary aim of this study was to develop a new model to predict the risk of HCC in CHB patients treated with ETV or TDF that is more effective in predicting the long-term risk of HCC compared with the previous models. The secondary aim was to suggest a modified stratification of HCC surveillance between patients with different HCC risk levels using the newly developed model.

## 2. Materials and Methods

### 2.1. Study Design & Population

This is a retrospective cohort study which was approved by the institutional review boards of the participating hospitals and waived the requirement for informed consent.

CHB patients who received antiviral therapy between January 2007 and December 2020 were included in the study of all of the following inclusion criteria were satisfied: (a) age ≥ 19 years, (b) history of visiting the liver clinic or admission to the hospital because of problems related to CHB, and (c) receiving treatment with ETV or TDF. Patients were excluded if one or more of the following exclusion criteria were indicated: (a) chronic kidney disease necessitating dialysis, (b) HCC or non-treatable malignant tumor coexisting at the initiation of antiviral therapy, (c) lack of data from essential examinations (liver imaging studies, such as ultrasonography or computed tomography or laboratory tests quantitating HBV DNA levels) at baseline, or (d) missing regular HCC surveillance examinations. Furthermore, patients without baseline liver stiffness data were excluded.

By reviewing the medical records of the patients and applying the criteria above, patients were selected from four university-affiliated hospitals in the Republic of Korea (Korea University Guro Hospital, Korea University Ansan Hospital, Gacheon University Gil Medical Center, Wonju Severance Christian Hospital). A total of 3827 patients were initially included in the entire cohort; however, many of these patients lacked data on liver stiffness, which was one of our primary interests in this study. After excluding patients who lacked data on liver stiffness, 1895 patients were finally selected as the study cohort. Demographic data, alcohol consumption, laboratory tests related to liver diseases, and history of liver-related complications were investigated through the review of medical records. Development of HCC was defined as the primary outcome, while death and liver transplantation were considered secondary outcomes. Serum alpha-fetoprotein measurement and ultrasonography were routinely performed every 6 months for HCC surveillance at all centers according to the practice guidelines [24].

The flowchart of the study process is shown in Supplementary Figure S1.

### 2.2. Definitions

Liver cirrhosis was diagnosed based on ultrasonographic findings including undulation of liver surfaces, blunt liver edge, severe coarse liver echotexture, liver shrinkage, splenomegaly, or ascites [16,25,26]. Patients were considered alcohol consumers if the consumption of alcohol was >40 g/week for males and >20 g/week for females based on the record from the initial visit, at which quantity (g/week), frequency (times/week), and duration (year) of alcohol consumption were assessed [27]. Virological response was defined as undetectable HBV DNA (<20 IU/mL) by real time PCR [24]. HCC was diagnosed according to the European Association for the Study of the Liver criteria [28].

### 2.3. Statistical Analysis

The study patients were randomly assigned to either the derivation or validation cohorts at a 2:1 ratio. For each cohort, categorical variables are presented as frequencies with percentages, while continuous variables are described as mean values with standard deviations or medians with interquartile ranges (IQRs).

We estimated a minimal sample size considering sensitivity and specificity with an alpha error of 0.05 and a precision of 0.1 [29]. Based on studies by Fan et al. and Kim et al. [13,30], sensitivity was set to 0.85, specificity to 0.60, and prevalence to 0.08. We found that the minimum sample size was 1153 (Supplementary Materials).

We performed a univariate analysis in the derivation cohort using time-dependent Cox regression analysis and confirmed the statistically significant variables. For continuous variables, we defined cut-off values that were clinically significant in predicting the risk of HCC considering the probability of the distribution and AUROC in the derivation cohort as well as the literatures [15,31,32]. Variables showing significance in univariate analyses were included in the multivariable analysis and assessed using Cox regression analysis. A backward-selection procedure was performed. Using variables that were confirmed to be statistically significant and independent, the model was developed in the derivation cohort. After developing the model, the regression coefficients from the final Cox proportional hazards model were transformed into scores. Cumulative risk scores were calculated for each patient, and time-varying receiver operating characteristic curves and the area under the receiver operating characteristic curves (AUROC) were assessed for the discrimination of the risk score. The AUROC test from the new model and other risk scores was performed using a nonparametric approach. The Kaplan–Meier method was used to estimate cumulative HCC incidence. Statistical analyses were performed using the Statistical Package for the Social Sciences (version 25; IBM SPSS Statistics Inc., Chicago, IL, USA) software. A two-sided *p*-value of < 0.05 was defined as statistically significant.

## 3. Results

### 3.1. Patient Baseline Characteristics

A total of 1895 patients were included in this study, and they did not significantly differ from 1932 patients who were excluded in terms of baseline characteristics, except for their antiviral agents. (Supplementary Table S1) After random allocation, there were 1239 patients in the derivation group and 656 patients in the validation group. The baseline characteristics of patients in the derivation and validation cohorts are shown in Table 1. There were no significant differences between the groups. The majority of patients were male (59.4% and 59.9%) and had a mean age of 47.72 ± 10.95 and 48.63 ± 10.92 years, respectively. At the baseline, 36.5% and 39.9% of the patients had cirrhosis, and 15.6% and 14.0% of the patients consumed alcohol, respectively. The median values of liver stiffness were 9.90 (6.10–17.30) and 9.95 (6.10–17.30) kPa, and those of alanine aminotransferase (ALT) were 87 (44–180) and 77 (44–160) U/L, respectively.

### 3.2. Antiviral Therapy

All the patients were treated with ETV or TDF continuously. Patients who stopped the medications were censored from the analysis. The virological response at the end of first year and 5th year was observed in 68.0% and 94.3% of the patients, respectively. Virological response rate was significantly higher in patients with liver cirrhosis than in non-cirrhotic patients at year 1 (73.6% vs. 64.3%, respectively, *p* < 0.001) and year 5 (95.9% vs. 93.1%, respectively, *p* = 0.002). Although virological response rate was lower in patients treated with ETV than in those with TDF at year 1 (65.2% vs. 73.5%, respectively, *p* < 0.001), it became similar at year 5 (94.0% vs. 94.9%, respectively, *p* = 0.34).

### 3.3. HCC Development

After starting antiviral agents, 165 patients were diagnosed as HCC on a median of 63.0 (IQR, 37.6–98.2) months with annual incidence of 1.5%. Among them, 75 (45.5%), 70 (42.4%), 17 (10.3%), and 3 (2.4%) were belonged to Barcelona Clinic Liver Cancer (BCLC) and had very early, early, intermediate, and advanced stages, respectively, at the diagnosis.

### 3.4. Model Development

Supplementary Table S2 shows the results of the univariate analysis in the derivation cohort. On univariate analysis, male sex, older age, excessive alcohol consumption, hypertension, diabetes mellitus (DM), and cirrhosis at baseline were significantly associated with the development of HCC. Laboratory results found to be potential predictors of HCC were thrombocytopenia (platelet count < 150,000/µL), hypoalbuminemia (albumin < 3.5 g/dL), ALT level < 80 U/L, total bilirubin ≥ 2.0 mg/dL, INR ≥ 1.7, and serum sodium level <140 mmol/L. In addition, AFP ≥ 10 ng/mL was found to be a potential predictor of HCC, and liver stiffness (<9.7, 9.7–14.9, ≥14.9) was a risk factor for HCC. Baseline HBV DNA, HBeAg, and antiviral agent (ETV or TDF) were not statistically significant in the univariate analysis. Model 1 was developed from significant predictors using the univariate analysis described above. In the multivariable analysis, the independent predictors were age [adjusted hazard ratio (aHR) = 1.771 (95% CI, 0.671–4.675) at 40–49 years; aHR = 3.682 (95% CI, 1.445–9.381) at ≥50 years; *p* < 0.0001], cirrhosis based on sonography at baseline [aHR = 2.974 (95% CI, 1.865–4.743), *p* <0.0001], consumption of alcohol (ethanol) [aHR = 1.954 (95% CI, 1.263–3.022, p = 0.003)], stiffness of the liver [aHR = 1.314 (95% CI, 0.728–2.371) at 9.7 ≤ LS < 14.9 kPa, aHR = 2.340 (95% CI, 1.440–3.804) at LS ≥ 14.9 kPa, *p* = 0.001], and normal to mildly elevated ALT (<80 U/L) [aHR = 1.633 (95% CI, 1.081–2.466), *p* = 0.002]. Model 2 was constructed using the variables mentioned above. The results of the multivariable analysis are presented in Table 2. Supplementary Figure S2 shows the cumulative risk of HCC by each variable using the Kaplan–Meier method.

### 3.5. Derivation of the ACCESS-HCC Score for Hepatocellular Carcinoma

Table 3 lists the beta coefficients for the five variables identified in model 2. The smallest beta-coefficient was 0.273 in liver stiffness of 9.7 ≤ LS < 14.9 kPa. The score for each variable is defined as the ratio of the beta-coefficient to 0.273. Thus, liver stiffness of 9.7 ≤ LS < 14.9 kPa was assigned one point, and the scores of other variables were assigned considering the ratio of the beta-coefficient. The scoring system named the ACCESS (age, cirrhosis, consumption of ethanol, stiffness of liver, serum alanine aminotransferase)-HCC prediction model is also shown in Table 3. The scores ranged from 0 to 14. The 3-year, 5-year, and 10-year risks of developing HCC projected for each are shown in Supplementary Table S3. For example, a total score of 0 had projected HCC risks of 0.3%, 0.9%, and 2.6% at 3, 5, and 10 years, respectively, whereas the corresponding risks for a score of 14 were 28.4%, 49.9%, and 93.6% at 3, 5, and 10 years, respectively.

### 3.6. Validation of the ACCESS-HCC Score

**Figure 1:**
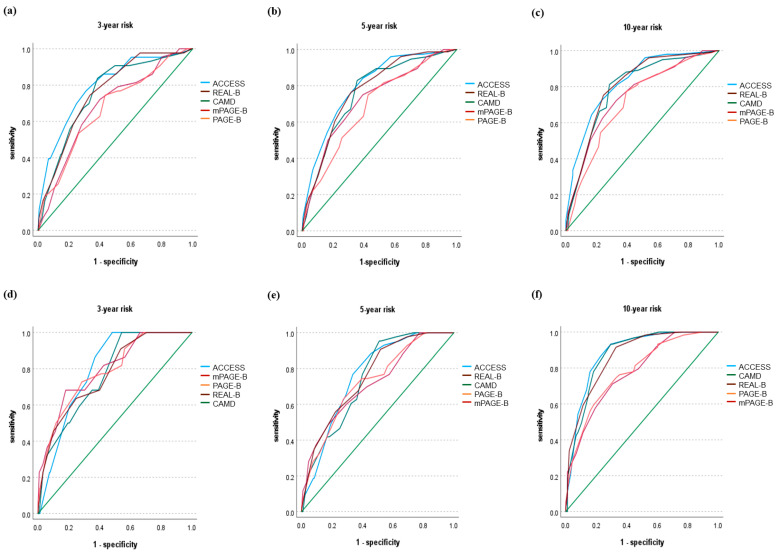
This is a figure Comparison of the AUROC in the ACCESS-HCC prediction model and the existing models from derivation cohort & validation cohort: (**a**) 3-year risk, (**b**) 5-year risk, (**c**) 10-year risk from derivation cohort; (**d**) 3-year risk, (**e**) 5-year risk, (**f**) 10-year risk from validation cohort.

Using the validation cohort, we produced AUROCs for the prediction of HCC risk at 3 years [AUROC = 0.798 (95% CI, 0.737–0.860)], 5 years [AUROC = 0.762 (95% CI, 0.701–0.824)], and 10 years [AUROC = 0.883 (95% CI, 0.830–0.937)]. In the same validation cohort, the ACCESS-HCC score had a better performance than the PAGE-B, mPAGE-B [30], REAL-B, and CAMD scores across all prediction years. (Figure 1 and Table 4) The ACCESS-HCC score performed especially better in predicting the 10-year risk of HCC compared with PAGE-B and mPAGE-B. Its performance for 10-year risk prediction was also better than that of the REAL-B and CAMD models, emphasizing that the ACCESS-HCC score is more effective in predicting the long-term risk of HCC than other models.

### 3.7. Risk Stratification Using the ACCESS-HCC Score

**Figure 2:**
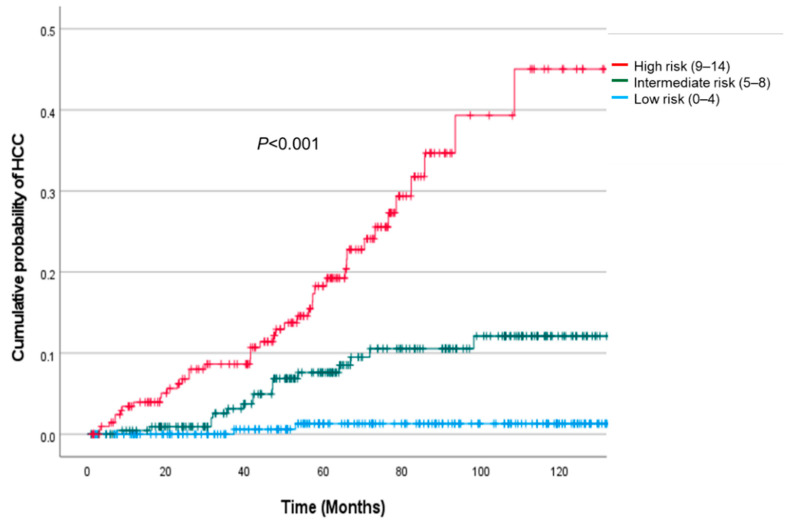
Cumulative incidence of hepatocellular carcinoma in the high-risk, intermediate-risk, and low-risk groups.

The cut-off values for the low-risk (0–4 points), intermediate-risk (5–8 points), and high-risk (9–14 points) groups were assigned according to the tertiles of the patients’ scores. A total of 214 (32.6%), 227 (34.6%), and 215 (32.8%) patients were classified into low-risk, intermediate-risk, and high-risk groups in the validation cohort, with 3, 17, and 40 HCC cases occurring during the 10 years of follow-up, respectively. The cumulative HCC incidence in the low-, intermediate-, and high-risk groups were analyzed using the Kaplan–Meier method (Figure 2). The estimated annual incidence rates by risk group using the 3-year data were 0.5% (95% CI, 0.1–0.9%), 3.2% (95% CI, 2.3–4.1%), and 11.3% (95% CI, 9.4–13.2%) in the low-risk, intermediate-risk, and high-risk groups, respectively. By comparing the estimated risk with the observed risk (Supplementary Table S4), we confirmed that the model had excellent performance in predicting the risk of HCC.

### 3.8. High-Risk Patients with Hepatocellular Carcinoma

During the follow-up of the high-risk group, 66 and 40 cases of HCC developed in the derivation and validation cohorts, respectively; over 70% of the high-risk patients had developed HCC within 5 years of the follow-up, and almost all of the high-risk patients had developed HCC within 10 years of the follow-up.

The mean ages of these HCC patients were 55.7 and 54.89 years at baseline in the derivation and validation cohorts, respectively. In total, 36.4% and 17.5% of the patients consumed alcohol, and 87.9% and 87.5% had cirrhosis at baseline, respectively. In addition, 74.2% and 80.0% had liver stiffness of ≥14.9 kPa and 80.3% and 77.5% had ALT less than 80 U/L, respectively. Hence, many risk factors were present simultaneously in this group of patients.

## 4. Discussion

In this retrospective cohort study of patients with CHB who were treated with ETV or TDF, we confirmed that the new ACCESS-HCC prediction model performed well in predicting the risk of HCC. The model performed better than previous models and was especially effective in predicting the long-term risk of HCC.

A new model was developed based on five variables (age, cirrhosis, consumption of alcohol, liver stiffness, and serum ALT). The data of these variables are routinely available in most clinical practices and can be obtained using noninvasive methods. The ACCESS-HCC model had a higher value of AUROC than those of PAGE-B and mPAGE-B. This model performed better than the other models in predicting the risk of HCC for five and ten years, indicating that the model has an advantage in predicting the long-term risk of HCC in CHB patients treated with antiviral agents. In addition, the ACCESS-HCC model included variables at baseline only, implying that the model can decrease the need for repeated examinations for risk prediction.

In this study, age was stratified into three: under 40, 40–49, and 50 years or over. This was done as HCC risk increases with increasing age above 40 years onwards, thus necessitating initiation of HCC surveillance. In those aged 50 years or over, surveillance is considered mandatory [33].

Liver cirrhosis based on ultrasonography at baseline is an important risk factor for HCC, and the fact that many HCC prediction models include cirrhosis as a variable supports this idea [14,15,16,18,19,21]. However, even in the absence of liver cirrhosis, the stage of liver fibrosis assessed by the degree of liver stiffness would influence HCC incidence. Nevertheless, most models lack liver stiffness as a variable [15,16,18,19,21]. The new ACCESS-HCC model has confirmed that liver stiffness is also an important assessment tool in HCC risk stratification and should be considered a variable. As there might be a multicollinearity between ultrasonographic liver cirrhosis and liver stiffness values, we assessed it by calculating variance influence factors, but the possibility was low.

The influence of ALT level in our model needs to be discussed. Unlike the previous model [21], HCC risk increases when ALT is normal to mildly increased (<80 U/L) in the ACCESS-HCC model. This is because CHB patients with advanced liver disease frequently show normal to mildly elevated ALT rather than high levels [34,35]. Hence, current clinical guidelines for HCC recommend initiating antiviral therapy in liver cirrhosis patients regardless of ALT levels and in CHB patients with significant liver fibrosis despite ALT being less than two times the upper limit of normal [33,36]. These findings iterate that antiviral therapy should not be delayed in CHB patients due to a limited elevation of ALT levels because the risk of HCC could be even higher in these patients.

HBV DNA levels were considered important in previous models, such as REACH-B [20]. However, in this study, it was shown that viral factors, including HBV, DNA, and hepatitis B e antigen, are no longer significant in predicting the risk of HCC. This could be attributed to the fact that current antiviral agents, such as ETV or TDF, effectively suppress HBV replication, with only a rare incidence of resistance. The type of antiviral agent was also not a significant factor for predicting development of HCC.

The importance of lifestyle-related risk factors, such as DM, obesity, and alcohol consumption, has recently increased [15,21]. In this study, alcohol consumption was selected as a significant variable. As alcohol consumption is a modifiable lifestyle factor that reduces the risk of HCC, strict abstinence should be emphasized.

In this study, the ACCESS-HCC model identified three distinct risk groups. The patients were divided into low-risk, intermediate-risk, and high-risk groups, and the estimated annual incidence of HCC was almost 50% at ten years in the high-risk group compared to less than 5% in the low-risk group. This suggests that more intensive surveillance is needed for high-risk patients. Currently, an ultrasound examination with or without serum alfa-fetoprotein assessment is used for HCC surveillance [28,33] but more detailed examinations are needed for these patients. In contrast, for low-risk patients, mitigating the current standards for surveillance can be considered to decrease the patient burden.

To summarize, the new model included five clinically important variables and performed well in terms of predicting the long-term risk of HCC using a simple formula. Stratified HCC surveillance is possible using the ACCESS-HCC prediction model in clinical practice.

Our study had several limitations. First, a portion of the initial cohort of 3827 patients lacked data on liver stiffness, which led to the exclusion of almost half of the patients. This was because these patients initiated antiviral treatment when liver stiffness measurements were not routinely conducted. However, the baseline characteristics of excluded patients were not much different from those of included patients. The key difference was the antiviral agent administered. Excluded patients had received ETV more often because TDF was available in Korea since 2012 (Supplementary Table S1), but the type of antiviral agents did not affect HCC development. Second, due to the inherent limitations of the retrospective study, the collection of information depended on medical records and might be inaccurate. The quantity and frequency of alcohol consumption particularly were mainly assessed at the time of the initial visit interview, and did not reflect changes in its quantity and frequency during the follow-up period. Third, our cohort included only Korean patients, which means that the results of the ACCESS-HCC model application in other international cohorts should be additionally evaluated. Finally, our cohort included patients treated with either ETV or TDF. However, newer antiviral agents, such as tenofovir alafenamide or besifovir, have been developed and are prescribed currently [33,36]; thus, validation in patients treated with these agents is needed.

In conclusion, we successfully developed the ACCESS-HCC score as a useful and reliable model for predicting the risk of HCC in CHB patients treated with antiviral agents. Further research is needed for external validation using larger cohorts with different characteristics.

## Figures and Tables

**Table 1:** Baseline characteristics of the derivation and validation cohorts.

Characteristics	Derivation Cohort (*n* = 1239)	Validation Cohort (*n* = 656)	*p* Value
	No. (%)	No. (%)	
Male sex	736 (59.4)	393 (59.9)	0.831
Age, mean (SD)	47.72 (10.95)	48.63 (10.92)	0.084
Antiviral agent			0.243
Entecavir	760 (61.7)	421 (64.5)	
Tenofovir	471 (38.3)	232 (35.5)	
HTN	161 (13.0)	74 (11.3)	0.282
DM	129 (10.4)	72 (11.0)	0.705
Alcohol drinking ^a^	193 (15.6)	92 (14.0)	0.184
CKD	33 (2.7)	26 (4.0)	0.121
Liver cirrhosis ^b^	452 (36.5)	262 (39.9)	0.140
Decompensation	105 (8.8)	57 (9.0)	0.918
Liver stiffness (kPa),^c^ median (IQR)	9.90 (6.10–17.30)	9.95 (6.10–17.30)	0.891
HBV DNA (IU/mL), median (IQR)	1.84 × 10^6^ (7.50 × 10^4^–3.26 × 10^7^)	1.88 × 10^6^ (6.87 × 10^4^–3.19 × 10^7^)	0.080
HBeAg	625 (51.6)	348 (54.5)	0.223
HBeAb	553 (46.7)	294 (47.6)	0.704
PLT × 10^3^/uL, median (IQR)	157 (114–210)	152 (113–201)	0.058
Albumin (g/dL), median (IQR)	4.1 (3.7–4.4)	4.1 (3.7–4.4)	0.401
ALT (U/L), median (IQR)	87 (44–180)	77 (44–160)	0.165
Total bilirubin (mg/dL), median (IQR)	0.90 (0.69–1.30)	0.90 (0.69–1.31)	0.602
INR, median (IQR)	1.08 (1.00–1.16)	1.07 (1.00–1.18)	0.435
Creatinine (mg/dL), median (IQR)	0.80 (0.60–0.90)	0.70 (0.68–0.90)	0.350
Na (mmol/L), mean (SD)	139.55 (2.88)	139.52 (2.82)	0.845
AFP (ng/mL), median (IQR)	5.01 (2.70–13.05)	5.00 (2.70–14.30)	0.904
Ascites	65 (5.3)	47 (7.2)	0.158
HEP ^d^			
1	1122 (99.7)	587 (100.0)	0.238
2	2 (0.2)	-	
3	1 (0.1)	-	

^a^ Alcohol consumption: >40 g/week for males, >20 g/week for females. ^b^ Liver cirrhosis was diagnosed based on ultrasonographic findings. ^c^ Liver stiffness was assessed by transient elastography. ^d^ HEP: (1) no hepatic encephalopathy; (2) West-Haven grade I or II; and (3) West-Haven grade III or IV. Abbreviations: PLT, platelet; ALT, alanine aminotransferase; INR, international normalized ratio; AFP, alpha-fetoprotein; HEP, hepatic encephalopathy.

**Table 2:** Multivariate Cox Regression Analysis for the independent predictors of hepatocellular carcinoma in the derivation cohort (*n* = 1239).

	Model 1		Model 2	
Variables	Multivariate-Adjusted Hazard Ratio (95% CI)	*p* Value	Multivariate-Adjusted Hazard Ratio (95% CI)	*p* Value
Age				
<40	Referent	<0.0001	Referent	<0.0001
40–49	1.762 (0.668–4.651)	0.253	1.771 (0.671–4.675)	0.248
≥50	3.557 (1.381–9.158)	0.009	3.682 (1.445–9.381)	0.006
HTN	1.276 (0.766–2.126)	0.350		
DM	1.225 (0.711–2.112)	0.465		
Alcohol ^a^	1.947 (1.247–3.041)	0.003	1.954 (1.263–3.022)	0.003
Liver cirrhosis ^b^	2.898 (1.792–4.687)	<0.0001	2.974 (1.865–4.743)	<0.0001
Liver stiffness ^c^				
<9.7 kPa	Referent	0.01	Referent	0.001
9.7 ≤ LS < 14.9 kPa	1.284 (0.710–2.323)	0.409	1.314 (0.728–2.371)	0.365
≥14.9 kPa	2.122 (1.268–3.551)	0.004	2.340 (1.440–3.804)	<0.0001
ALT < 80	1.696 (1.119–2.573)	0.013	1.633 (1.081–2.466)	0.020
PLT < 150,000	0.926 (0.584–1.468)	0.745		
Alb < 3.5	1.174 (0.751–1.834)	0.482		
AFP ≥ 10	1.292 (0.849–1.965)	0.232		

^a^ Alcohol consumption: >40 g/week for males, >20 g/week for females. ^b^ Liver cirrhosis was diagnosed based on ultrasonographic findings. ^c^ Liver stiffness was assessed by transient elastography. Abbreviations: HTN, hypertension; DM, diabetes mellitus; ALT, alanine aminotransferase; PLT, platelet; Alb, albumin; AFP, alpha-fetoprotein.

**Table 3:** Derivation of the ACCESS prediction score for hepatocellular carcinoma using the derivation cohort (*n* = 1239).

Parameter	Beta-Coefficient	ACCESS Score
Age		<40: 0
	0.572	40–49: 2
	1.304	≥50: 4
Alcohol ^a^		No: 0
	0.670	Yes: 2
Liver cirrhosis ^b^		No: 0
	1.090	Yes: 3
Liver stiffness ^c^		<9.7 kPa: 0
	0.273	9.7 ≤ LS < 14.9 kPa: 1
	0.850	≥14.9 kPa: 3
ALT		≥80: 0
	0.490	<80: 2

^a^ Alcohol consumption: >40 g/week for males, >20 g/week for females. ^b^ Liver cirrhosis was diagnosed based on ultrasonographic findings. ^c^ Liver stiffness was assessed by transient elastography. Abbreviations: LS, liver stiffness; ALT, alanine aminotransferase.

**Table 4:** Areas under the ROC Curves for predicting the 3-, 5-, and 10-year hepatocellular carcinoma risk using ACCESS and other scores in the validation cohort (*n* = 656).

Prediction Model	Time-Dependent AUROC (95% CI)		
	3-Year Risk Prediction	5-Year Risk Prediction	10-Year Risk Prediction
ACCESS	0.798 (0.737–0.860)	0.762 (0.701–0.824)	0.883 (0.830–0.937)
PAGE-B	0.787 (0.695–0.879)	0.725 (0.649–0.802)	0.782 (0.707–0.856)
mPAGE-B	0.797 (0.706–0.889)	0.720 (0.638–0.801)	0.777 (0.702–0.851)
REAL-B	0.772 (0.680–0.864)	0.757 (0.689–0.825)	0.867 (0.811–0.923)
CAMD	0.770 (0.689–0.852)	0.747 (0.685–0.810)	0.874 (0.819–0.930)

Abbreviations: ACCESS, age-cirrhosis-consumption of ethanol-stiffness of liver-serum alanine aminotransferase; PAGE-B, patient’s age-gender-platelets score; mPAGE-B, modified PAGE-B; REAL-B, real-world effectiveness from the Asia Pacific rim, liver consortium for HBV score; CAMD, cirrhosis-age-male sex-diabetes mellitus.

## Data Availability

Data are available on request to the corresponding authors.

## Supplementary Materials

The following supporting information can be downloaded at: https://www.mdpi.com/article/10.3390/jcm11226613/s1, Supplementary Methods: Sample size calculation; Figure S1: Study process flowchart; Figure S2: Kaplan–Meier graphs of hepatocellular carcinoma according to the statistically significant and independent variables; Table S1: Baseline characteristics of the included and excluded patients; Table S2: Incidence Rate and Univariate Analysis of the Potential Predictors of Hepatocellular Carcinoma in the Derivation Cohort (*n* = 1239); Table S3: Projected Hepatocellular Carcinoma Risk for the ACCESS score; Table S4: Model-based Risk of Hepatocellular Carcinoma by Actual Cumulative Incidence.
