# Supplementary material for: Long-Term Prediction Model for Hepatocellular Carcinoma in Patients with Chronic Hepatitis B Receiving Antiviral Therapy: Based on Data from Korean Patients
Source: J Clin Med. 2022 Nov 8;11(22):6613. doi: 10.3390/jcm11226613 (PMC9697157; doi:10.3390/jcm11226613)
Supplement: Supplementary file 1 [file jcm-11-06613-s001.zip › jcm-1968231-supplementary.pdf]

Supplementary Methods

The sample size was calculated considering the sensitivity and specificity of the model (Buderer, 1996) [1].

The following formula was adopted for estimation.

$$n_{Sn} = \frac{z^2\alpha/2 \; Sn\,(1 - Sn)}{d^2 \times Pv}$$

$$n_{Sp} = \frac{z^2\alpha/2 \; Sp\,(1 - Sp)}{d^2 \times (1 - Pv)}$$

→ $n = \max(n_{Sn},\ n_{Sp}),\ Y = 1 \text{ vs } Y = 0$

$n_1 = n \times P_v,\ n_2 = n \times (1 - P_v)$

- Assumptions for calculation were

  → alpha(α) = 0.05, precision(d) = 0.1,

  → sensitivity(S_n) = 0.85, Specificity(S_p) = 0.60, prevalence(P_v) = 0.08

- Sample size estimation

$$n_{Sn} = \frac{1.959964^2 \times 0.85 \times (1 - 0.85)}{0.1^2 \times 0.08} = 613$$

$$n_{Sp} = \frac{1.959964^2 \times 0.60 \times (1 - 0.60)}{0.1^2 \times 0.08} = 1{,}153$$

→ $n = \max(n_{Sn},\ n_{Sp})$ = 1,153

Reference

1. Buderer, N.M. Statistical methodology: I. Incorporating the prevalence of disease into the sample size calculation for sensitivity and specificity. *Acad. Emerg. Med.* **1996**, *3*, 895–900.

Supplementary Table S1. Baseline characteristics of the included and excluded patients.

Characteristics	Included Patients (n = 1895)	Excluded Patients (n = 1932)	*p* Value
	No. (%)	No. (%)	
Male sex	1129 (59.6)	1179 (61.0)	0.378
Age, mean (SD)	48.04 (10.94)	49.39 (12.11)	0.063
Antiviral agent			0.038
Entecavir	1181 (62.3)	1353 (70.0)	
Tenofovir	714 (37.7)	579 (30.0)	
HTN	235 (12.4)	285 (14.8)	0.068
DM	201 (10.6)	242 (12.5)	0.071
Alcohol drinking [a]	285 (15.0)	278 (14.4)	0.308
CKD	59 (3.1)	78 (4.0)	0.147
Liver cirrhosis [b]	721 (38.1)	793 (41.0)	0.062
Decompensation	162 (8.9)	201 (10.4)	0.078
Liver stiffness (kPa),[c] median (IQR)	9.90 (6.10-17.30)	9.30 (6.20-16.00)	0.550
HBV DNA (IU/mL), median (IQR)	1.87×10^{6} (7.11×10^{4}-3.22×10^{7})	1.83×10^{6} (5.88×10^{4}-2.30×10^{7})	0.242
HBeAg	973 (51.3)	966 (54.5)	0.458
HBeAb	851 (47.1)	822 (47.8)	0.704
PLT$\times10^{3}$/uL, mean (SD)	164.3 (70.1)	150.1 (75.2)	0.066
Albumin (g/dL), mean (SD)	3.99 (0.56)	3.79 (0.62)	0.052
ALT (U/L), mean (SD)	84 (44-171)	71 (37-139)	0.062
Total bilirubin (mg/dL), median (IQR)	0.90 (0.69-1.30)	1.00 (0.70-1.50)	0.072
INR, median (IQR)	1.127 (0.241)	1.204 (0.420)	0.088
Creatinine (mg/dL), mean (SD)	0.898 (0.870)	0.936 (1.143)	0.629
Na (mmol/L), mean (SD)	139.5 (2.9)	138.8 (3.3)	0.106
AFP (ng/mL), mean (SD)	44.3 (241.4)	49.4 (340.6)	0.613
Ascites	112 (6.0)	171 (8.9)	0.064
HEP[d]			0.480
1	1890 (99.7)	1926 (99.6)	
2	3 (0.2)	3 (0.2)	
3	2 (0.1)	3 (0.2)	

[a]Alcohol consumption: >40 g/week for males, >20 g/week for females. [b]Liver cirrhosis was diagnosed base on ultrasonographic findings. [c]Liver stiffness was assess by transient elastography. [d]HEP: (1) no hepatic encephalopathy; (2) West-Haven grade I or II; and (3) West-Haven grade III or IV. Abbreviations: PLT, platelet; ALT, alanine aminotransferase; INR, international normalized ratio; AFP, alpha-fetoprotein; HEP, hepatic encephalopathy.

Supplementary Table S2. Incidence rate and univariate analysis of the potential predictors of hepatocellular carcinoma in the derivation cohort (n=1,239)

Variable	Category	No. of HCC Cases	Hazard Ratio (95% CI)	*p* Value
Sex	Female	36	Referent	
	Male	69	1.295 (0.865–1.939)	0.209
Age	<40	5	Referent	
	40–49	28	3.120 (1.205–8.080)	0.019
	≥50	72	7.201 (2.908–17.828)	<0.0001
Antiviral agent	Entecavir	70	Referent	
	Tenofovir	35	0.961 (0.637–1.450)	0.850
HTN	No	85	Referent	
	Yes	20	1.786 (1.096–2.908)	0.020
DM	No	88	Referent	
	Yes	17	1.794 (1.066–3.019)	0.028
Alcohol	No	76	Referent	
	Yes	29	2.177 (1.418–3.341)	<0.0001
CKD	No	101	Referent	
	Yes	4	1.384 (0.509–3.759)	0.524
Liver cirrhosis	No	29	Referent	
	Yes	76	5.425 (3.531–8.336)	<0.0001
Decompensation	No	86	Referent	
	Yes	12	1.546 (0.845–2.828)	0.158
Liver stiffness (kPa)	LS<9.7	26	Referent	
	9.7≤LS<14.9	21	2.062 (1.160–3.665)	0.014
	LS≥14.9	58	3.768 (2.372–5.985)	<0.0001
HBV DNA	Negative	6	Referent	
	Positive	99	0.704 (0.308–1.606)	0.404
HBeAg	Negative	57	Referent	
	Positive	46	0.685 (0.464–1.010)	0.056
HBeAb	Negative	50	Referent	
	Positive	52	1.359 (0.921–2.006)	0.122
PLT (/uL)	$\geq 150\times 10^3$	34	Referent	
	$<150\times 10^3$	71	2.483 (1.649–3.737)	<0.0001
Albumin (g/dL)	≥3.5	75	Referent	
	<3.5	30	2.241 (1.467–3.423)	<0.0001
ALT (U/L)	≥80	39	Referent	
	<80	66	2.360 (1.587–3.510)	<0.0001
Total bilirubin (mg/dL)	<2	87	Referent	
	≥2	18	1.469 (0.884–2.441)	0.138
INR	<1.7	95	Referent	
	≥1.7	5	1.556 (0.633–3.826)	0.335
Creatinine (mg/dL)	<1.5	6	Referent	
	≥1.5	0	0.770 (0.107–5.522)	0.795
Na (mmol/L)	≥140	32	Referent	
	<140	35	1.542 (0.954–2.492)	0.077
AFP (ng/mL)	<10	58	Referent	
	≥10	47	1.936 (1.317–2.846)	<0.0001
Ascites	No	95	Referent	
	Yes	10	1.909 (0.995–3.663)	0.052
HEP	1	101	Referent	
	2	0	Unmeasurable	0.970
	3	1	6.938 (0.965–49.887)	0.054

Abbreviations: HTN, hypertension; DM, diabetes mellitus; CKD, chronic kidney disease; PLT, platelet; ALT, alanine aminotransferase; INR, international normalized ratio; AFP, alpha-fetoprotein; HEP, hepatoencephalopathy

Supplementary Table S3. Projected hepatocellular carcinoma risk for the ACCESS score

Score	HCC Risk		
	3-Year	5-Year	10-Year
0	0.3%	0.9%	2.6%
1	0.4%	1.3%	4.0%
2	0.6%	1.8%	6.1%
3	0.8%	2.5%	9.3%
4	1.1%	3.4%	13.9%
5	1.6%	4.7%	20.2%
6	2.2%	6.5%	28.4%
7	3.2%	8.8%	38.4%
8	4.5%	11.9%	49.4%
9	6.3%	15.8%	60.6%
10	8.7%	20.8%	70.7%
11	12.0%	26.8%	79.1%
12	16.3%	33.8%	85.6%
13	21.7%	41.7%	90.3%
14	28.4%	49.9%	93.6%

Abbreviations: ACCESS, age-cirrhosis-consumption of ethanol-stiffness of liver serum alanine aminotransferase

Supplementary Table S4. Model-based risk of hepatocellular carcinoma by actual cumulative incidence

Score	3-Year Estimated	3-Year Observed	5-Year Estimated	5-Year Observed	10-Year Estimated	10-Year Observed
Low (0–4) N = 115	0.1%–0.9%	Not observed	0.5%–1.7%	Not observed	2.6%–7.6%	Not observed-
Intermediate (5–8) N = 230	2.3%–4.1%	2.6%	7.3%–10.9%	8.4%	32.0%–42.4%	28.4%
High (9–14) N = 172	9.4%–13.2%	9.3%	21.9%–28.1%	21.5%	66.7%–76.1%	76.9%

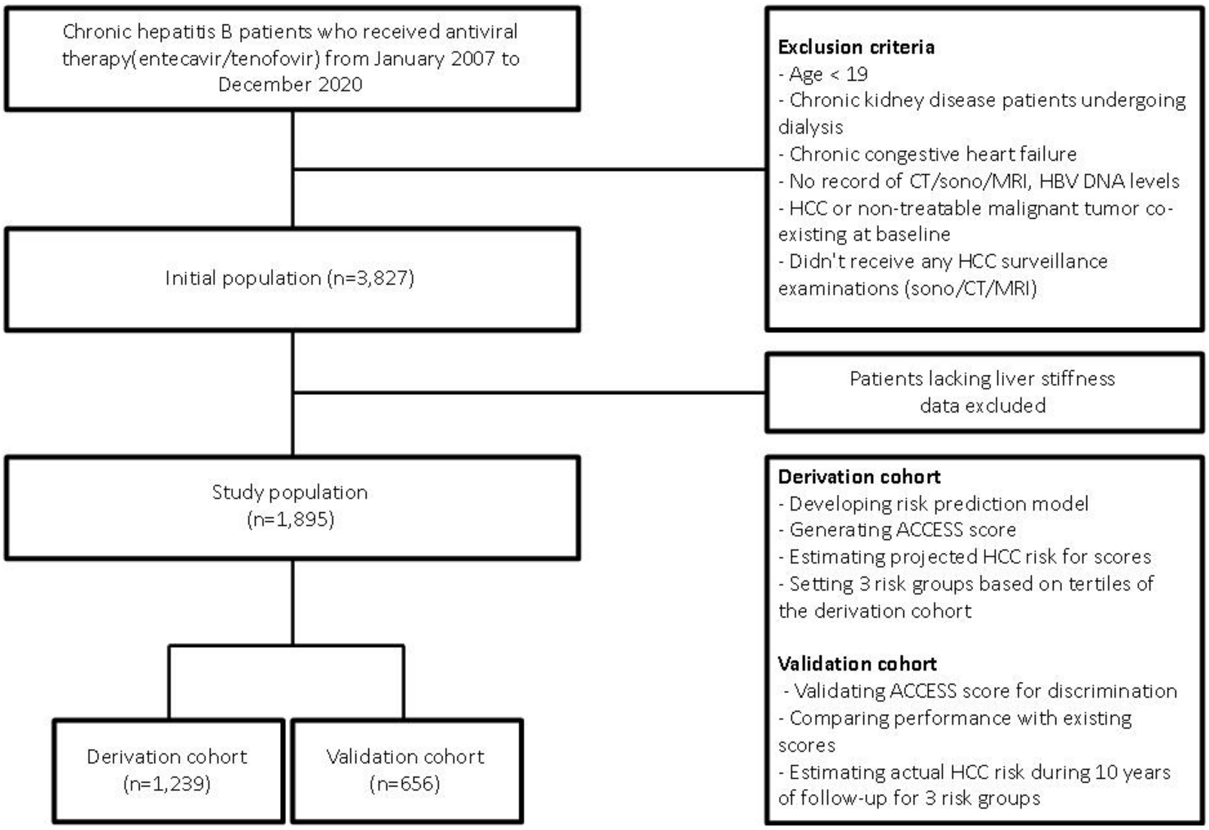

Supplementary Figure S1. Study process flowchart.
Abbreviations: HCC, hepatocellular carcinoma; ACCESS, age-cirrhosis-consumption of ethanol-stiffness of liver-serum ALT

(**a**)

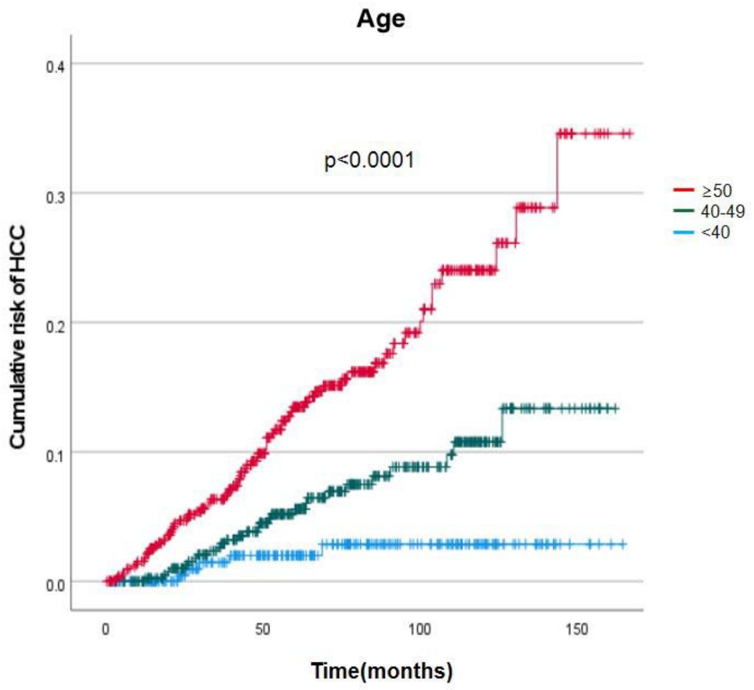

(**b**)

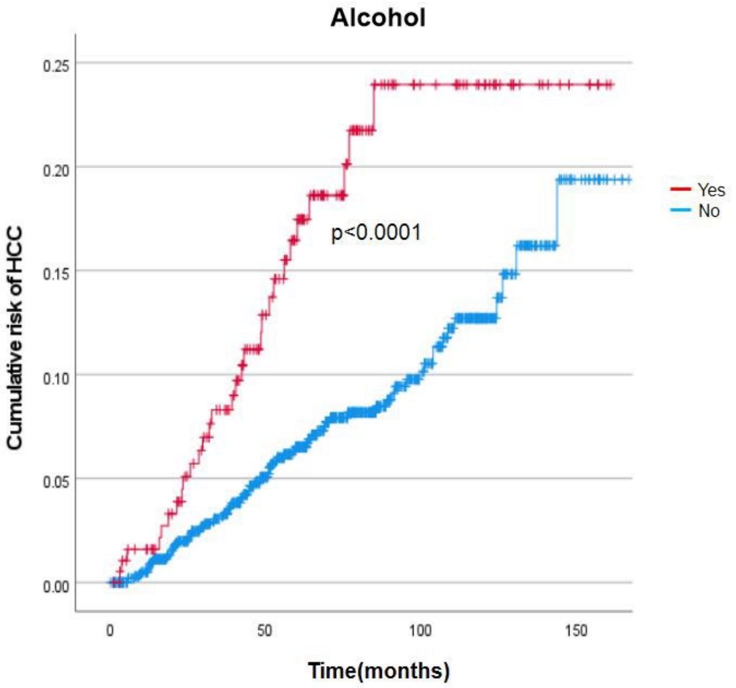
Alcohol
Cumulative risk of HCC
0.25
0.20
0.15
0.10
0.05
0.00
0
50
100
150
Time(months)
p<0.0001
Yes
No

(**c**)

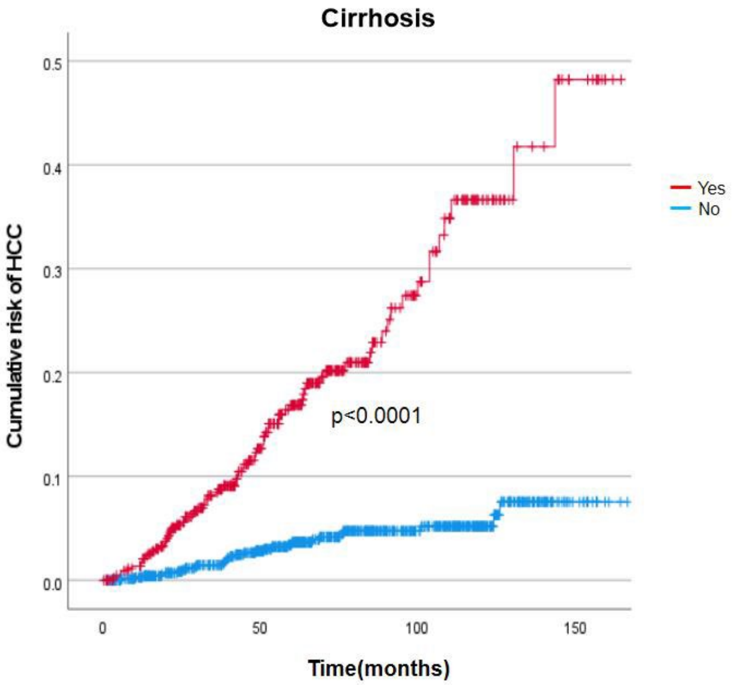
Cirrhosis
Cumulative risk of HCC
0.5
0.4
0.3
0.2
0.1
0.0
0
50
100
150
Time(months)
p<0.0001
Yes
No

(**d**)

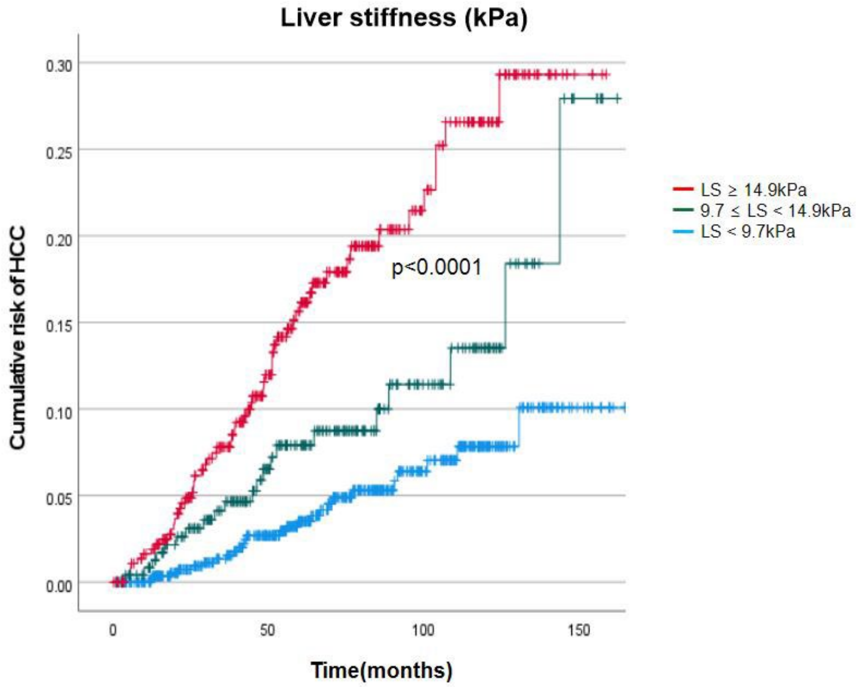

(**e**)

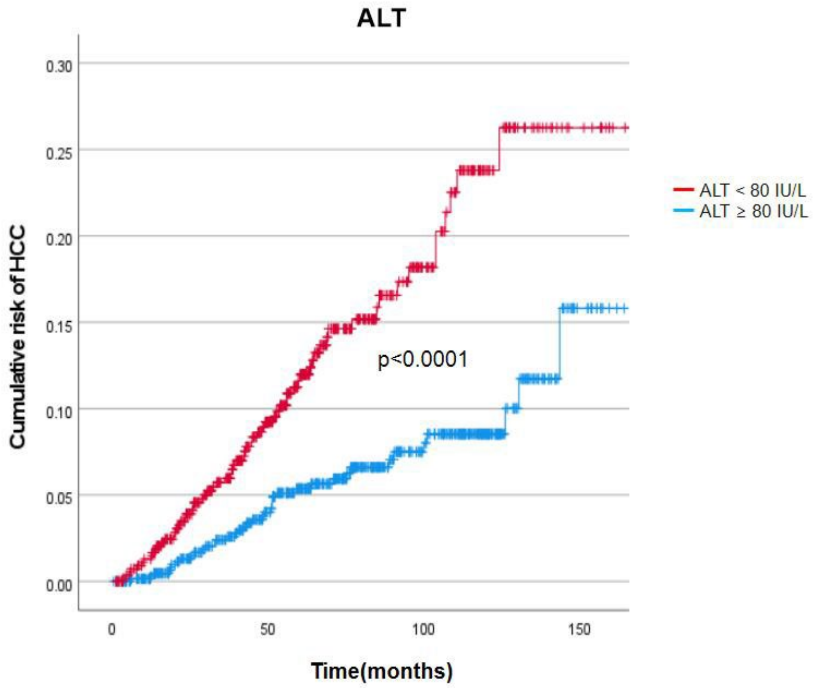

Supplementary Figure S2. Kaplan–Meier graphs of hepatocellular carcinoma according to the statistically significant and independent variables. (**a**) age, (**b**) alcohol, (**c**) liver cirrhosis, (**d**) liver stiffness, and (**e**) alanine aminotransferase
